# Comparative evaluation of Sensititre YeastOne and CLSI M38-Ed3 reference method for determining echinocandin minimum effective concentrations against *Aspergillus* isolates

**DOI:** 10.1128/spectrum.00280-24

**Published:** 2024-08-20

**Authors:** Assaf Potruch, Hila Elinav, Matan J. Cohen, Alexander Rouvinski, Itzhack Polacheck, Maya Korem

**Affiliations:** 1 Faculty of Medicine, Hebrew University of Jerusalem, Jerusalem, Israel; 2 Department of Nephrology and Hypertension, Hadassah Medical Center, Jerusalem, Israel; 3 Department of Microbiology and Infectious Diseases, Hadassah Medical Center, Jerusalem, Israel; 4 Clalit Health Services, Jerusalem, Israel; 5 Department of Microbiology and Molecular Genetics, Institute for Medical Research Israel-Canada, Hebrew University-Hadassah Medical School, Jerusalem, Israel; 6 The Kuvin Center for the Study of Infectious and Tropical Diseases, Hebrew University of Jerusalem, Jerusalem, Israel; University of Maryland School of Medicine, Baltimore, Maryland, USA

**Keywords:** minimum effective concentration, echinocandin, *Aspergillus*, paradoxical effect, Sensititre YeastOne, broth microdilution

## Abstract

The minimum inhibitory concentration (MIC) of echinocandins against *Aspergillus* spp. does not represent the actual inhibition threshold of echinocandins. Therefore, the recommended method to evaluate their activity is determining the minimum effective concentration (MEC) in broth microdilution, a method that is less common in clinical settings. This study aimed to assess a user-friendly commercial method, Sensititre YeastOne (SYO), to determine the effectiveness of echinocandins (caspofungin, anidulafungin and micafungin) against *Aspergillus* spp. Echinocandins MEC was determined against 23 isolates of *Aspergillus* spp. using SYO and the reference Clinical and Laboratory Standards Institute (CLSI) method. MECs were read with an inverted microscope and a reading mirror. Essential agreement (EA) between the tested methods was defined as a ±twofold dilution difference. There was a high EA (91%–100%) between the reference method and SYO in determining echinocandins MEC against *Aspergillus* isolates using inverted microscopy. A high EA was also observed between SYO MEC determined by inverted microscopy and a reading mirror, but different incubation times were required. SYO is a reliable, simple method for determining the MEC of echinocandins against *Aspergillus* isolates, preferably with an inverted microscope, and can be easily used in clinical laboratories when echinocandin susceptibility testing is required.

**IMPORTANCE:**

Using a commercial method such as Sensititre YeastOne (SYO) to determine the minimum effective concentration (MEC) of echinocandins against *Aspergillus* spp. has been shown to be a reliable alternative to the Clinical and Laboratory Standards Institute (CLSI) reference method. This makes it more suitable for high-volume clinical laboratories. SYO provides accurate results comparable to the standard method and could potentially improve patient care by guiding more optimal antifungal treatment choices for patients with *Aspergillus* infections.

## INTRODUCTION

Invasive aspergillosis (IA) is a severe mold infection caused by *Aspergillus* species that affects mainly immunocompromised patients, with an incidence of 15%–20% among neutropenic patients with hematologic malignancies and is associated with a fatality rate as high as 20%–60%, despite antifungal therapy (1, 2). The recommended primary therapy is voriconazole, a triazole inhibiting fungal cytochrome P450-mediated 14-alpha lanosterol demethylation, which causes cell membrane damage. Echinocandins can be used as salvage therapy when multiple drug-drug interactions or adverse effects limit voriconazole use (3). Increasing azole resistance of *Aspergillus* isolates is another major concern requiring the consideration of other antifungals (4).

Echinocandins inhibit glucan synthase, a membrane enzyme, required to synthesize β-1,3-glucan in the fungal cell wall. The loss of β-1,3-glucan induces cell wall remodeling of *Aspergillus* hyphae, resulting in short, rounded, and compact hyphae but not inhibition of its growth. This effect is usually observed at low concentrations (<0.03 µg/mL) for most *Aspergillus* spp., thus defining a minimal effective concentration (MEC), instead of an actual minimum inhibitory concentration (MIC) (5). Consequently, the MEC is used instead of MIC to indicate the lowest concentration of drug causing hyphal damage, manifested as small, rounded, compact hyphae formation as seen in the broth microdilution (BMD) susceptibility test standardized by the Clinical and Laboratory Standards Institute (CLSI) (6). Echinocandins MEC can be read visually following a BMD susceptibility test using a reading mirror (6) or an inverted microscope (7). However, since the CLSI reference BMD method is comprehensive and requires an expert and laborious testing process, it is not commonly used in high-volume clinical laboratories.

Sensititre YeastOne (SYO) is a BMD-based commercial system that uses a colorimetric indicator (AlamarBlue) to assess the effectiveness of antifungals on yeasts and molds (Image S1) (8). This method is user-friendly and was previously compared to the CLSI reference method to determine MICs of echinocandins in the treatment of *Candida* spp. and consequently validated (9, 10).

This study aimed to compare SYO and the CLSI M38 3rd edition reference method for determining the MEC of echinocandins against *Aspergillus* spp, using a reading mirror and an inverted microscope.

## MATERIALS AND METHODS

### Isolates

A total of 23 clinical and reference isolates of *Aspergillus* spp. stored at −80°C at the Hadassah microbiology laboratory were thawed, grown on Sabouraud Dextrose Agar (SDA, Novamed, Israel) at 30°C and re-identified morphologically and by the matrix-assisted laser desorption/ionization-time of flight mass spectrometry (MALDI-TOF MS, bioMerieux, Marcy-l 'Etoile, France). The isolates included 10 *Aspergillus fumigatus*, 9 *Aspergillus flavus*, 3 *Aspergillus terreus*, and 1 *Aspergillus ochraceus*. Two of the *A. fumigatus* isolates were reference strains: *A. fumigatus* DPL 1035 (a caspofungin-resistant isolate) and *A. fumigatus* ATCC MYA-3626 (anidulafungin MEC 0.015 µg/mL within range of ≤0.016 µg/mL according to CLSI M38M51S) (11).

### CLSI BMD susceptibility testing

BMD susceptibility testing was performed according to the CLSI reference M-38Ed3 method with twofold decreasing concentrations of micafungin, anidulafungin, and caspofungin (6). After 24 h of incubation at 35°C, all BMD plates were inspected under an inverted microscope (Nikon Eclipse TS100, Melville, NY) to determine MEC. Plates were also examined with a reading mirror after 24 and 48 h of incubation to determine MEC.

### SYO susceptibility testing

Commercially available SYO (YO10) plates (Thermo Scientific, UK) were used according to the manufacturer’s recommendations (12). SYO plates were incubated at 35°C and examined at 24 and 48 h using an inverted microscope and a reading mirror to determine MEC, respectively.

All antifungal susceptibility testing in the study was performed in duplicates. The experiment was repeated twice at different times for reproducibility assessment.

### Definitions

**FIG 1:**
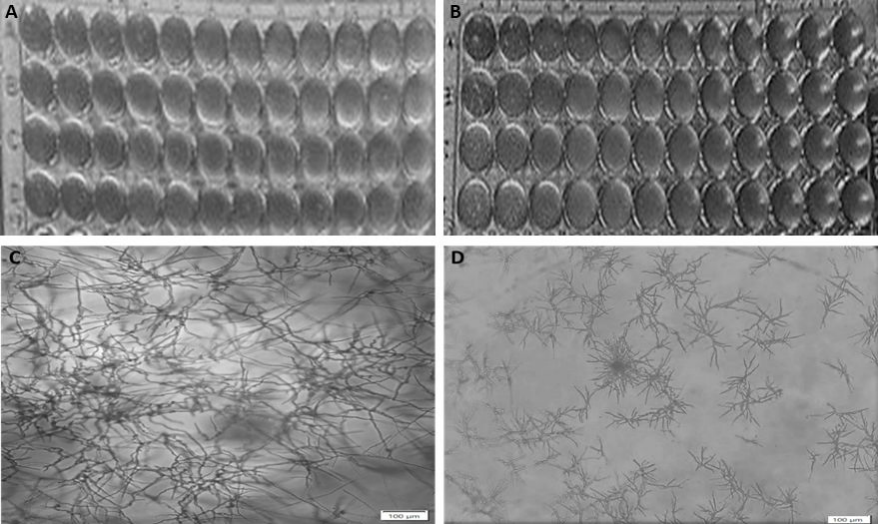
Determination of MEC of caspofungin against *A. fumigatus*. (A and B) BMD according to the CLSI methodology with four *A. fumigatus* including ATCC MYA-3626 (row A) and clinical isolates (row B–D), visualized via a reading mirror after 24 h (A) and 48 h (B) of incubation. Columns 1–10 contain drug concentrations that decrease in twofold steps from 4 to 0.008 µg/mL and columns 11–12 are controls. MEC was difficult to read at 24 h but was determined as 0.0625 µg/mL in row A and 0.5 µg/mL in rows B–D after 48 h of incubation. (C and D) An example of SYO BMD wells visualized in an inverted microscope (×10) following incubation of ATCC MYA-3626, without antifungals (C—normal hyphal growth), and following 24 h incubation with caspofungin at 0.008 µg/mL (D—multibranched rosettes representing MEC).

MEC is the lowest concentration of an echinocandin resulting in the growth of small, rounded, compact hyphal forms compared with the hyphal growth seen in the growth control well using a reading mirror (6) or the formation of multibranched rosettes seen with an inverted microscope (7) (Fig. 1).

CLSI BMD results were considered the reference standard. Essential agreement between the tested methods was defined as a ±2-fold dilution difference in MEC.

### Statistical analysis

All isolates were tested in duplicates at two different time points. Thus, each isolate was tested by each method (reference BMD and SYO) four times, referred to as four runs.

We compared the median minimum effective concentration (MEC) of each echinocandin against all *Aspergillus* species (spp.) combined, using the SYO and the reference BMD method, with results analyzed using an inverted microscope. This comparison was performed for each run, and a pooled median for all cumulative runs (Tables 1 and 2, respectively) was calculated. We conducted a subgroup analysis for each *Aspergillus* spp. separately to compare the SYO and reference BMD methods (Table S1).

**TABLE 1:** Comparison of the SYO and CLSI BMD methods to determine the MEC of echinocandins against *Aspergillus* spp. by inverted microscopy

Antifungal agent	Run^*a*^	MEC (µg/mL) of 23 isolates by inverted microscopy		*P* value	Essential agreement (±2-fold dilution difference) (%)
		CLSI BMD MEC median (range)	SYO MEC median (range)		
Anidulafungin	1	0.015 (0.008–1)	0.015 (0.015–0.5)	0.502	95
	2	0.015 (0.008–1)	0.015 (0.015–0.5)	0.166	100
	3	0.015 (0.008–1)	0.015 (0.015–0.5)	0.545	100
	4	0.015 (0.008–1)	0.015 (0.015–0.5)	0.959	100
Caspofungin	1	0.015 (0.008–0.5)	0.025 (0.008–0.5)	0.673	91
	2	0.015 (0.008–0.5)	0.025 (0.008–0.5)	0.866	91
	3	0.015 (0.008–1)	0.015 (0.008–0.5	0.859	95
	4	0.015 (0.008–1)	0.015 (0.008–1)	0.893	95
Micafungin	1	0.015 (0.008–0.5)	0.015 (0.008–1)	0.396	100
	2	0.015 (0.008–1)	0.015 (0.008–1)	0.436	100
	3	0.015 (0.008–1)	0.015 (0.008–1)	0.608	100
	4	0.015 (0.008–1)	0.015 (0.008–1)	0.964	100

^*a*^ Runs 1, 2 and 3, 4 are duplicates. Each run included 23 *Aspergillus* isolates.

**TABLE 2:** Comparison of echinocandin-*Aspergillus* cumulative MEC, determined by inverted microscopy, between SYO and reference BMD methods

Antifungal	MEC (µg/mL) of 92 isolates^*a*^ determined by inverted microscopy		*P-*value
	CLSI BMD MEC median (range)	SYO MEC median (range)	
Anidulafungin	0.015 (0.008–2)	0.015 (0.015–2)	0.342
Caspofungin	0.015 (0.008–8)	0.0225 (0.008–8)	0.948
Micafungin	0.015 (0.008–2)	0.0115 (0.008–4)	0.545

^*a*^ Cumulative 92 isolates of 4 runs (46 duplicate isolates from 2 different time points).

Additionally, in each run, we compared the median MEC of all *Aspergillus* isolates between two methods: an inverted microscope after 24 h of incubation and a reading mirror after 48 h of incubation. The time gap was necessary due to the lack of well-developed compact hyphal forms observed in the reading mirror at the 24-h timepoint (Table 3 and Fig. 1).

**TABLE 3:** Comparison of the MEC of echinocandins against *Aspergillus* spp. using inverted microscopy and a reading mirror following SYO antifungal testing

Antifungal	Run^*a*^	SYO MEC (µg/mL)		*P*-value	Agreement (%)
		Inverted microscope, following 24 h incubation, median (range)	Reading mirror following 48 h incubation, median (range)		
Anidulafungin	1	0.015 (0.015–0.5)	0.015 (0.008–1)	0.138	95
	2	0.015 (0.015–0.5)	0.015 (0.008–1)	0.063	95
	3	0.015 (0.015–0.05)	0.015 (0.015–1)	0.24	95
	4	0.015 (0.015–0.5)	0.015 (0.015–1)	0.113	95
Caspofungin	1	0.015 (0.008–1)	0.015 (0.008–2)	0.207	100
	2	0.015 (0.008–1)	0.015 (0.008–2)	0.173	100
	3	0.015 (0.008–1)	0.015 (0.008–1)	0.307	95
	4	0.015 (0.008–1)	0.015 (0.008–1)	0.596	95
Micafungin	1	0.0225 (0.008–0.5)	0.015 (0.008–2)	0.04	87
	2	0.0225 (0.008–0.5)	0.015 (0.008–2)	0.092	87
	3	0.015 (0.008–0.5)	0.015 (0.008–1)	0.327	91
	4	0.015 (0.008–1)	0.015 (0.008–1)	0.137	91

^*a*^ Runs 1, 2 and 3, 4 are duplicates.

The essential agreement was calculated by determining the number of test results within the ±2-fold dilution of the MEC determined by the reference method.

Comparisons were performed using the paired signed Wilcoxon rank test.

Statistical analysis was performed using SPSS version 29 (IBM Corp., Armonk, NY); a *P*-value of 0.05 was considered statistically significant.

## RESULTS

All strains (*n* = 23) showed significant growth in control wells in both methods after 24 h incubation.

**FIG 2:**
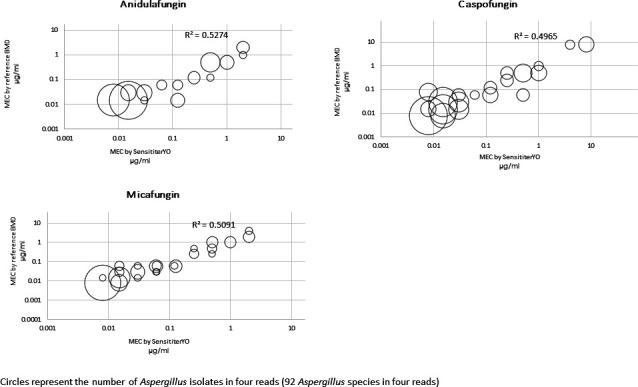
Comparison of SYO and reference CLSI methods in determining MEC of three echinocandins against *Aspergillus* spp.

Median MEC of the three echinocandins (caspofungin, anidulafungin, and micafungin) against *Aspergillus* isolates, read with an inverted microscope, were similar in the reference BMD and SYO methods when examined for each run or all run together (Tables 1 and 2 and Fig. 2).

The median MEC of each echinocandin was determined by inverted microscopy against six groups of *Aspergillus* spp. (Eight *A. fumigatus*, nine *A. flavus*, three *A. terreus*, one *A. ochraceus*, and two reference DPL 1035 and ATCC MYA-3626 *A. fumigatus*). Comparing SYO and reference BMD, the essential agreement was 100% for *A. flavus*, *A. terreus*, *A. ochraceus*, and the *A. fumigatus* DPL 1035 and ATCC MYA-3626 strains. For *A. fumigatus* clinical isolates, the essential agreement between echinocandins was 100% for micafungin, 94% for caspofungin, and 84% for anidulafungin, with significantly lower median MEC reads of caspofungin in SYO compared to the reference BMD (*P* < 0.001, Table S1).

SYO MEC determined by inverted microscopy following 24 h of incubation compared to the reading mirror following 48 h of incubation revealed a high essential agreement (≥95%) for both anidulafungin and caspofungin. Agreement was lower for micafungin (87%–91%) (Table 3). A similar comparison of MEC determined by inverted microscopy and reading mirror according to the reference BMD method was not performed due to the absence of clear compact hyphal forms in the reading mirror after 48 h of incubation.

No statistical difference was found when each run was compared to the other three in determining SYO MEC of echinocandins against *Aspergillus* spp., confirming the reproducibility of the test.

## DISCUSSION

This study is the first to evaluate the use of SYO combined with inverted microscopy or a reading mirror to determine the susceptibility of different *Aspergillus* isolates to echinocandins compared to the reference CLSI BMD.

The results indicate that SYO provides reliable MEC values with up to a twofold dilution difference compared to the reference BMD. This implies that SYO can substitute the BMD, which is currently considered the reference gold standard test, especially when limitations hinder routine BMD application.

The recommended primary therapy for IA is voriconazole. Echinocandins are second-line therapy and are used in patients who are refractory to primary azole therapy (3), partially due to the high MIC sometimes observed upon *Aspergillus* incubation with different echinocandin preparations (13). Nevertheless, when exposed to echinocandins, the normal growth of the mold is disrupted and may be observed as short and compact hyphae. Thus, in cases of adverse events or drug-drug interactions that exclude treatment with first-line therapy with azoles, echinocandins can be used alternatively (3) following evaluation of echinocandins MEC (5) according to the CLSI methodology (6). However, former studies have pointed to a low reproducibility of the CLSI method in determining echinocandins MEC (14). SYO is a validated laboratory method to assess the susceptibility of yeasts to different antifungal preparations. Former comparisons of the reference BMD method with SYO evaluated antifungal susceptibility measured as MIC of *Candida* species, *Cryptococcus neoformans*, and *Aspergillus* species to amphotericin B, itraconazole, posaconazole, and voriconazole, and yielded promising results with excellent agreement rates (15–17). Nevertheless, the evaluation of echinocandins MIC against *Aspergillus* spp. using SYO color endpoints (18) yielded a significant variance in the results.

We compared the CLSI reference method and the readily used SYO to determine the MEC based on the appearance of small, rounded, compact hyphal forms or multibranched rosettes using a reading mirror and an inverted microscope.

The reference BMD or SYO required 36–48 h of incubation to develop dense hyphal structures that could be observed macroscopically using the reading mirror. There was no difference in MECs using SYO determined by the reading mirror or inverted microscopy. However, a 24-h incubation was sufficient to determine echinocandin MECs with the inverted microscope, as it is more sensitive for detecting small changes in hyphal formation. Shorter incubation enables an earlier and more accurate reading of the MEC and adjustment of appropriate antifungal treatment for patients with IA. However, an inverted microscope is not available in all mycology laboratories.

Most *Aspergillus* spp. lack clinical breakpoints to antifungals. Clinical breakpoints, based on clinical trial data, susceptibility surveillance, resistance mechanism, and pharmacological parameters of antimicrobials, are used to predict the resistance or susceptibility of a specific fungal isolate to an antifungal agent (19). As most of these data and parameters do not exist for molds, epidemiological cut-off values (ECVs) are used to identify isolates with decreased susceptibility (6, 19). The ECV defines the upper limit of susceptibility for the wild-type (WT) population of isolates. Until now, ECVs were determined by the CLSI for caspofungin against *A. fumigatus* (0.5 µg/mL), *A. flavus* (0.5 µg/mL), *A. terreus* (0.12 µg/mL) and *Aspergillus niger* (0.25 µg/mL). The European Committee on Antimicrobial Susceptibility Testing (EUCAST) does not publish ECVs for echinocandins against *Aspergillus* species because of limited data (20). According to the CLSI ECVs (21, 22), the MEC of caspofungin against all nine isolates of *A. flavus* was comparable with WT strains (<0.5 µg/mL); 3/10 *A*. *fumigatus,* including the caspofungin resistant (control) isolate DPL 1035, were non-WT (possibly resistant) (>0.5 µg/mL), and the other seven were below the ECV. The two *A. terreus* included in this study were WT (<0.12 µg/mL). When read with the inverted microscope, these results were similar in the reference CLSI and SYO BMD. Therefore, we propose that the SYO can accurately determine the susceptibility of *Aspergillus* isolates to caspofungin for which ECVs are available (as mentioned above, the essential agreement for caspofungin between the methods is at least 91%, Table 1).

The main limitations of the study are (i) the MEC readings by a mirror at 48 h instead of 24 h of incubation as recommended by the reference CLSI, due to the lack of easily visualized compact hyphal forms at 24 h time point, though readings in the inverse microscope at 24 h were comparable between the methods and (ii) the lack of clinical breakpoints for echinocandins against *Aspergillus* spp. and the absence of ECVs for echinocandins other than caspofungin against *Aspergillus* spp., making the interpretation of the data and correlation of MEC with clinical outcomes, difficult.

Nevertheless, acquired echinocandin resistance among *Aspergillus* spp. is a rare event as the cost of *FKS1* target gene mutations may be an important loss of fitness (23).

In conclusion, SYO is a reliable, simple, and reproducible method for determining the MEC of echinocandins against *Aspergillus* isolates, preferably using an inverted microscope. Although a reading mirror can also be used, in this study it required a longer incubation time. Further studies, including additional *Aspergillus* isolates, are needed to ascertain the exact conditions for the SYO use to determine susceptibility to echinocandins.

## SUPPLEMENTAL MATERIAL

Supplemental table and image spectrum.00280-24-s0001.docx Table S1 and Image S1.

ASM does not own the copyrights to Supplemental Material that may be linked to, or accessed through, an article. The authors have granted ASM a non-exclusive, world-wide license to publish the Supplemental Material files. Please contact the corresponding author directly for reuse.
