# Supplementary material for: Comparative evaluation of Sensititre YeastOne and CLSI M38-Ed3 reference method for determining echinocandin minimum effective concentrations against Aspergillus isolates
Source: Microbiol Spectr. 2024 Aug 20;12(10):e00280-24. doi: 10.1128/spectrum.00280-24 (PMC11449228; doi:10.1128/spectrum.00280-24)

Supplemental material

Title: Comparative evaluation of Sensititre YeastOne and CLSI M38-Ed3 reference method for determining echinocandin minimum effective concentrations against *Aspergillus* isolates

Supplementary Table 1: Subgroup comparison of echinocandin-*Aspergillus* minimum effective concentration by inverted microscopy between SYO and CLSI BMD, according to *Aspergillus* species.

| Isolate (number) | Antifungal | | CLSI BMD MEC median, µg/mL (range) | SYO MEC median, µg/mL (range) | | Agreement (%) |
| --- | --- | --- | --- | --- | --- | --- |
|  |  | |  |  | |  |
| *A. fumigatus (8)* | ANID | | 0.1875 (0.015-1) | 0.09 (0.015-0.5) | | 84 |
|  | CAS | | 0.12 (0.008-1) | 0.06 (0.008-1) | | 94 |
|  | MCF | | 0.1225 (0.015-1) | 0.06 (0.015-1) | | 100 |
| *A. flavus (9)* | ANID | | 0.015 (0.008-0.5) | 0.015 (0.015-0.5) | | 100 |
|  | CAS | | 0.015 (0.008-0.5) | 0.015 (0.008-1) | | 100 |
|  | MCF | | 0.008 (0.008-1) | 0.008 (0.008-1) | | 100 |
| *A. terrerus (3)* | ANID | | 0.0115 (0.008-0.015) | 0.015 (0.015-0.03) | | 100 |
|  | CAS | | 0.0225 (0.008-0.03) | 0.0115 (0.008-0.06) | | 100 |
|  | MCF | | 0.008 (0.008-0.008) | 0.008 (0.008-0.008) | | 100 |
| *A. ochraceus (1)* | ANID | | 0.015 (0.015-0.015) | 0.015 (0.015-0,015) | | 100 |
|  | CAS | | 0.008 (0.008-0.008) | 0.008 (0.008-0.008) | | 100 |
|  | MCF | | 0.0115 (0.008-0.015) | 0.008 (0.008-0.008) | | 100 |
| *A. fumigatus* DPL 1035 (1) | ANID | | 2 | 2 | | 100 |
|  | CAS | | 8 | 8 | | 100 |
|  | MCF | | 2 | 2 | | 100 |
| *A. fumigatus* ATCC MYA-3626 (1) | ANID | | 0.008 | 0.015 | | 100 |
|  | CAS | | 0.008 | 0.008 | | 100 |
|  | MCF | 0.008 | | 0.008 | 100 | |

CLSI, Clinical and Laboratory Standards Institute; BMD, broth microdilution; MEC, minimum effective concentration; SYO, Sensititre YeastOne; ANID, anidulafungin; CAS, caspofungin; MCF; micafungin.

Supplementary Image 1. Sensititre YeastOne plate

A Sensititre YeastOne 10 plate incubated with *Aspergillus fumigatus* ATCC MYA-3626. Rows contain increasing two-fold concentrations of antifungal agents. After incubation, the results are read visually. The lowest concentration of antifungal agent that inhibits fungal growth, as evidenced by the absence of a color change from blue to pink, is the minimum inhibitory concentration (MIC). Well A1 serves as the growth control, containing no antifungal agent.


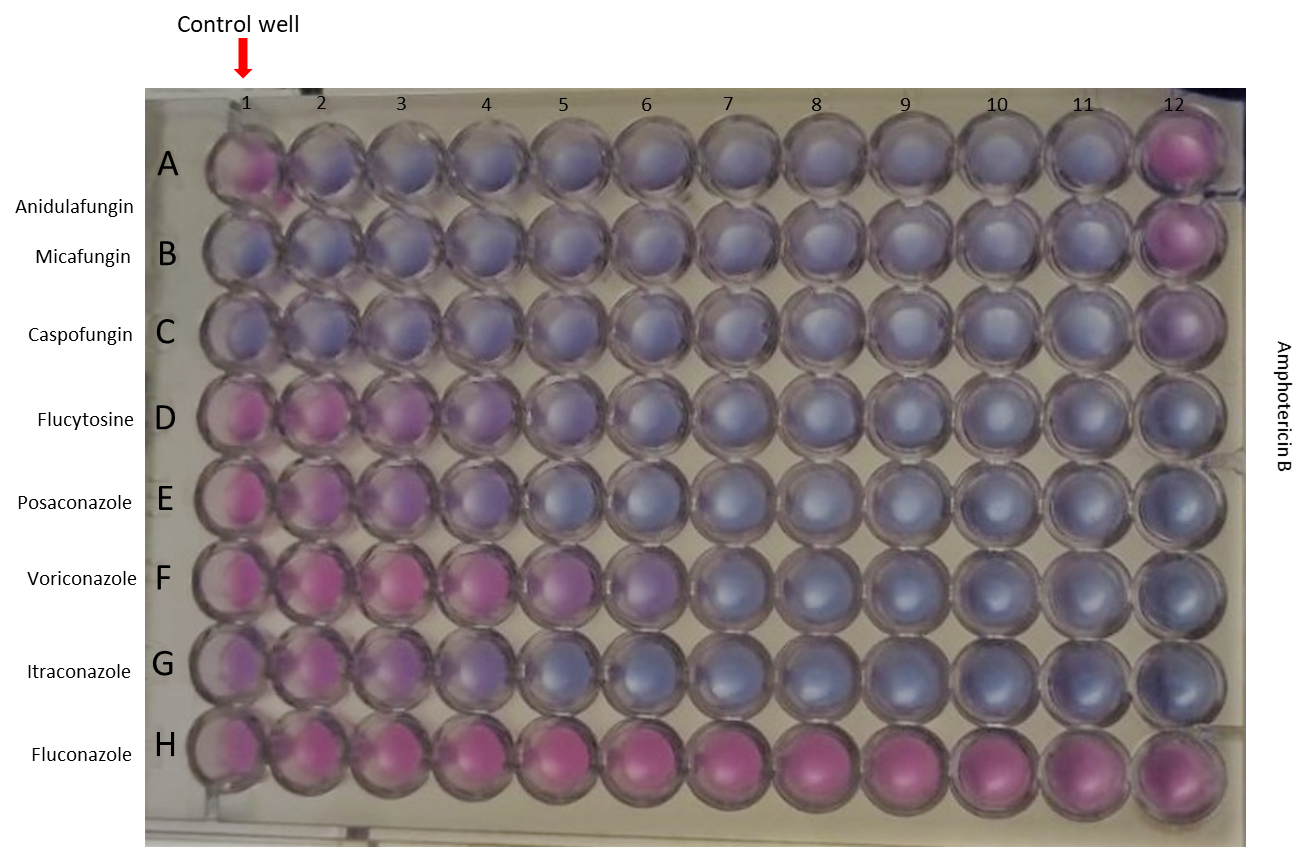

Supplement: Supplemental table and image — Table S1 and Image S1. [file spectrum.00280-24-s0001.docx]
